# Characterization of Silver Nanoparticles Synthesized Using *Hypericum perforatum* L. and Their Effects on *Staphylococcus aureus*

**DOI:** 10.1002/jemt.24862

**Published:** 2025-03-23

**Authors:** Canan Sevinc‐Sasmaz, Fatih Erci, Emrah Torlak, Mustafa Yöntem

**Affiliations:** ^1^ Department of Biotechnology, Faculty of Science Necmettin Erbakan University Konya Turkey; ^2^ Department of Molecular Biology and Genetics, Faculty of Science Necmettin Erbakan University Konya Turkey; ^3^ Department of Nursing, Faculty of Health Sciences Karamanoglu Mehmetbey University Karaman Turkey

**Keywords:** antibacterial, antibiofilm, green synthesis, *S. aureus*, silver nanoparticles

## Abstract

This study investigates the synthesis of silver nanoparticles (AgNPs) using *Hypericum perforatum* L. and evaluates their antibacterial and antibiofilm activities against *Staphylococcus aureus* . The synthesized AgNPs were characterized by UV‐Vis spectroscopy, X‐ray diffraction (XRD), transmission electron microscopy (TEM), and Fourier‐transform infrared spectroscopy (FTIR). UV‐Vis spectroscopy showed a maximum absorption peak at 448 nm, which indicates that nanoparticles have been formed successfully. TEM analysis showed that the AgNPs were spherical, with an average size of 35 ± 2.7 nm. FTIR confirmed the presence of functional groups on the surface of AgNP that may be contributing to its biological activity. The AgNPs exhibited significant antibacterial activity, with a minimum inhibitory concentration (MIC) of 75 μg/mL and an inhibition zone of 13 ± 0.13 mm at this concentration. They were also highly effective in inhibiting biofilm formation even at a concentration of 25 μg/mL, reducing biofilm formation by 47.25% ± 3.51%. At increased concentrations, nanoparticles have been shown to compromise bacterial membranes, leading to significant membrane disruption. This disruption subsequently results in a reduction of cellular respiration, with observed decreases of approximately twofold when compared to controls. Additionally, nanoparticles facilitate the production of superoxide anions, which can rise by about threefold, consequently enhancing the overall effectiveness of bacterial inactivation. Field emission scanning electron microscopy (FE‐SEM) revealed structural damage to bacterial cells treated with AgNPs, supporting their antimicrobial effects. These findings suggest that AgNPs synthesized from *H. perforatum* could serve as effective antimicrobial agents against *S. aureus* . Their ability to disrupt bacterial cell membranes, inhibit respiration, and induce oxidative stress makes them promising candidates for antimicrobial and antibiofilm applications, particularly given the increasing concern over bacterial resistance to conventional antibiotics.

## Graphical abstract

This study investigated the effects of biosynthesized silver nanoparticles (AgNPs) derived from *Hypericum perforatum* on the cell morphology of Gram‐positive *Staphylococcus aureus* bacterial cells, with FE‐SEM analysis showing distinct morphological changes in AgNP‐treated *S. aureus* cells compared to the control group.

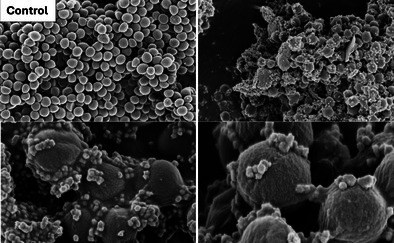

**Summary**

- AgNPs synthesized from *Hypericum perforatum* L. disrupt bacterial membranes, inhibit respiration, and generate superoxide anions.
- Notably, AgNPs showed significant antibiofilm activity at the MIC concentration (75 μg/mL), reducing biofilm formation by 83.20%.

## 1 Introduction

All materials from 1 to 1000 nm are referred to as nanomaterials, but nanoparticles between 1 and 100 nm have distinct physical, chemical, and biological characteristics compared to larger particles. This size range offers significant advantages in various applications due to a high surface‐to‐volume ratio, chemical stability, high catalytic reactivity, tunable pore size, enhanced mechanical strength, and antibacterial properties (Dolai et al. 2021).

Nanoparticles can be synthesized using two primary approaches: the bottom‐up and top‐down methods. The bottom‐up approach involves assembling smaller particles into larger structures, often utilizing chemical methods for this process. In contrast, the top‐down approach focuses on breaking down larger particles into nanoscale dimensions through physical methods (Kumari et al. 2023). Recent advancements have introduced environmentally sustainable methods to counter the harmful side effects typically associated with chemical and physical nanoparticle synthesis. These “greener” approaches have garnered increasing attention due to their potential to reduce environmental impact. Green nanotechnology offers a promising alternative by facilitating the synthesis of eco‐friendly nanoparticles, while minimizing both environmental degradation and risks to human health commonly linked to traditional nanomaterial production (Nasrollahzadeh et al. 2019).

The antibacterial properties of silver have been widely researched and applied in fields like medicine, food packaging, and industry (Elfaig et al. 2020; Li et al. 2024). Silver is incorporated into creams, dressings, and food packaging films to prevent contamination and treat burns and ulcers (Castellano et al. 2007; Tong 2009; Li et al. 2024). Green synthesized silver nanoparticles (AgNPs) are among the most valuable metallic nanoparticles, prized for their strong antimicrobial properties, making them highly effective in medical applications aimed at infection control and able to combat various microorganisms (Erci and Torlak 2019; Bruna et al. 2021). The exact biocidal mechanisms of nanoparticles are still under investigation, but they are likely to involve membrane disruption, protein denaturation, growth inhibition, enzyme inactivation, and morphological changes (McGivney et al. 2017; Bharti et al. 2024). For silver nanoparticles, the primary mechanism is the release of silver ions, which bind to sulfur‐containing proteins in the membrane and cell wall, causing rupture and increasing permeability. Silver ions also disrupt respiratory enzymes, leading to ROS production and ATP disruption (Feng et al. 2000; Klueh et al. 2000; Yin et al. 2020). Beyond their well‐known antimicrobial properties, green synthesized AgNPs have also shown significant pharmaceutical and biological potential, including analgesic, anticoagulant, and antihyperlipidemic activities (Barabadi, Mobaraki, et al. 2023; Barabadi, Noqani, et al. 2023; Barabadi et al. 2024). In addition to silver nanoparticles, green synthesis approaches have been widely explored for other metal‐based nanoparticles, including zinc oxide, copper, gold, titanium dioxide, iron oxide, and selenium (Malik et al. 2023).

*S. aureus* has emerged as a significant public health issue due to its increasing virulence and resistance to a growing number of antibiotics. One of its key virulence factors is its ability to form biofilms, which play a crucial role in the development of medical device‐associated infections. In recent years, trauma surgery departments worldwide have reported a substantial rise in *S. aureus* biofilm infections linked to medical implants, with biofilms also being detected in 93.5% of chronic wounds. *S. aureus* bacteria embedded in biofilms can be 10–1000 times more resistant to antibiotics than their free‐floating counterparts. Several antibiotics, including aminoglycosides, are ineffective in preventing bacterial biofilm formation and may even promote it (Costerton et al. 1999; Hoffman et al. 2005).

Recent studies highlight the use of various plant extracts for green AgNP synthesis (Chung et al. 2016; Marslin et al. 2018). *H. perforatum* (St. John's Wort), a medicinal plant from the *Hypericum* genus, is known for its bioactive compounds, such as hypericin, hyperforin, and flavonoids, which make it an ideal candidate for nanoparticle synthesis (Alahmad et al. 2021). In this study, AgNPs were synthesized using a simple, nontoxic method with aqueous extract of *H. perforatum* without additives or stabilizers, offering an environmentally friendly approach. The formation of AgNPs was confirmed by UV‐Vis, Fourier‐transform infrared spectroscopy (FTIR), and X‐ray diffraction (XRD) analyses, with size and shape characterized by TEM. The antibacterial and antibiofilm effects of AgNPs were tested against *S. aureus* , as well as their impact on cellular functions such as respiration, membrane permeability, cell morphology, and superoxide generation.

## 2 Materials and Methods

### 2.1 Green Synthesis of Silver Nanoparticles

*Hypericum perforatum* was collected during the flowering season, between June and July, from Konya province. The plant was washed three times with distilled water and dried at room temperature. Then, 200 mL of distilled water was added to 10 g of the plant sample, and the mixture was stirred for 2 h at 60°C. The resulting extract was filtered and stored at 4°C until use. To synthesize AgNPs, 10 mL of the extract was mixed with 90 mL of a 1 mM AgNO_3_ (Sigma‐Aldrich Chemicals, St Louis, MO, USA) solution and stirred at 60°C. AgNP formation was monitored by observing the color change, and the synthesized nanoparticles were centrifuged at 10,000 rpm for 15 min at 4°C. The supernatant was discarded, the pellet was washed twice with distilled water, and finally lyophilized to obtain nanoparticle powder.

### 2.2 Characterization of AgNPs

The optical properties of AgNPs were characterized by recording UV‐Vis spectra (Cary 60, Agilent Technologies, Santa Clara, CA, USA) over a wavelength range of 200–800 nm. The crystal structure of the nanoparticles was determined using XRD (Empyrean, PANalytical BV, Netherlands), with the XRD pattern analyzed between 10° and 80° in the 2θ range and a step size of 0.02. Biomolecules associated with the nanoparticles in the synthesis were identified through FTIR (Thermo Scientific, Waltham, MA, USA), with samples scanned in the infrared range of 400–4000 cm^−1^. The size, shape, and morphology of AgNPs were characterized using transmission electron microscopy (TEM 1400, JEOL, Tokyo, Japan).

### 2.3 Antibacterial Activity of AgNPs

The antibacterial activity of AgNPs was evaluated using microdilution and agar well diffusion assays, along with a growth curve assay to assess the time‐ and concentration‐dependent effects on the bacteria tested. Lyophilized culture of *S. aureus* (ATCC 25923) was supplied by Microbiologics Inc. (Saint Cloud, MN, USA), and stock cultures were stored at −80°C in tryptic soy broth (TSB; Neogen, Lansing, MI, USA) with 15% glycerol. A cell suspension of the microorganism was prepared at a density of approximately 1.5 × 10^8^ colony‐forming units (CFU)/mL in TSB and used as inoculum in in vitro antibacterial assays.

A standard microdilution method was used to determine the minimum inhibitory concentration (MIC) and minimum bactericidal concentration (MBC) of AgNPs. Each well of a sterile microplate was filled with 130 μL of fresh Mueller Hinton medium (MHB; Merck, Darmstadt, Germany) and 20 μL of diluted inoculum (10^6^ CFU/mL), followed by 50 μL of twofold diluted NP solutions, resulting in final concentrations ranging from 2 to 1000 μg/mL. Control wells without NPs and negative controls containing only NPs were included. After 24 h of incubation at 37°C, cell growth was assessed by measuring the optical density (OD) at 600 nm using a microplate reader (Multiskan Go; Thermo Scientific). The MIC is defined as the lowest NP concentration at which no visible bacterial growth is observed. A volume of 0.01 mL from each well was then inoculated onto Mueller Hinton agar (MHA; Merck) plates to determine the MBC, defined as the lowest concentration at which no colonies formed after incubation (Kızılkonca et al. 2021).

The well diffusion assay was performed on MHA plates with 10 mm diameter wells. A volume of 100 μL of inoculum was spread on the agar plates, and 50 μL of AgNP solutions at concentrations of 100, 75, 50, and 25 μg/mL were added to the wells. After incubation at 37°C for 24 h, the clear inhibition zones around the wells were measured in mm. The antibacterial activity of the nanoparticles was evaluated by comparing the inhibition zone diameters with those of gentamicin (10 μg, Oxoid, Thermo Fisher Scientific, Basingstoke, UK) disks (Balouiri et al. 2016).

The time‐ and concentration‐dependent effects of AgNPs were evaluated by growing test bacteria in a 96‐well microplate, following a modified method by Niyonshuti et al. (2020). The wells were filled with 180 μL of medium supplemented with increasing concentrations of NPs, ranging from 25 to 100 μg/mL. Then, 20 μL of diluted inoculum (10^6^ CFU/mL) was added to each well, bringing the final volume to 200 μL. Wells without NPs served as controls, with AgNO_3_ as a positive control. Negative controls containing only NPs were also included. The plate was incubated at 37°C, and absorbance at 600 nm was measured at regular intervals over 16 h using a microplate reader.

### 2.4 Antibiofilm Activity of AgNPs

The biofilm production of *S. aureus* ATCC 25923 was verified using the Congo red agar (CRA) method, as described by Arciola et al. (2006). *Escherichia coli* ATCC 25922 served as a biofilm‐negative control. Strains were inoculated onto CRA plates and incubated at 37°C for 24 h, followed by overnight incubation at room temperature. Biofilm‐positive *S. aureus* formed black colonies, while biofilm‐negative *E. coli* formed red colonies.

The antibiofilm activity of AgNPs was evaluated in 96‐well polystyrene microplates using a modified crystal violet method (Bianchini Fulindi et al. 2023). *S. aureus* was cultured and diluted 100‐fold in TSB medium with 2% glucose. The wells were filled with 180 μL of sterile medium, 10 μL of diluted culture, and 10 μL of AgNP solutions to final concentrations of 25, 50, 75, and 100 μg/mL. After 24 h of incubation at 37°C, the wells were washed three times with PBS to remove non‐adherent cells and air‐dried. The biofilm was stained with 150 μL of 0.1% crystal violet (Merck) for 30 min at room temperature. After washing three times with distilled water, 150 μL of 30% acetic acid was added, incubated for 10 min, and OD was measured at 595 nm. Wells without AgNPs served as the control, and wells with sterile TSB with 2% glucose served as the blank. Biofilm inhibition activity was calculated based on the average OD values.

$$\%\text{Biofilm inhibition}=\frac{\text{control}\;\mathrm{OD}-\text{test}\;\mathrm{OD}}{\text{control}\;\mathrm{OD}}x\;100$$

### 2.5 Effect of AgNPs on *S. aureus* Cellular Respiration

Respiration inhibition was evaluated using a modified method with 2,3,5‐triphenyl‐tetrazolium chloride (TTC) salt (Wahab et al. 2013). An overnight culture of *S. aureus* was inoculated into fresh TSB and incubated at 37°C for 4 h. After centrifugation at 5000 rpm for 10 min, the bacterial cells were washed with sterile PBS (pH 7.0) and resuspended to achieve an OD of 0.5 at 600 nm. Then, 150 μL of the cell suspension was transferred to a 96‐well microplate, and 40 μL of AgNPs was added to achieve concentrations of 25, 50, 75, and 100 μg/mL. Wells without nanoparticles served as controls, and the negative control contained only nanoparticles. To each well, 40 μL of 0.5% TTC (Sigma‐Aldrich) reagent was added, and the plate was incubated for 30 min in the dark. After incubation, the conversion of colorless TTC to red formazan was measured at 450 nm.

### 2.6 Effect of AgNPs on *S. aureus* Cell Membrane Permeability

Cell membrane permeability was assessed by measuring β‐galactosidase activity using o‐nitrophenyl‐β‐D‐galactopyranoside (ONPG; Sigma‐Aldrich) as a substrate (Han et al. 2013). *S. aureus* was cultured in TSB with 2% lactose, and the cell density was adjusted to 10^8^ CFU/mL. AgNPs (75 μg/mL) were added, and bacterial cells were grown to mid‐logarithmic phase (OD600: 0.4–0.6). After centrifugation, cells were resuspended in 20 mM sodium phosphate buffer (pH 7.5) with 100 mM NaCl, adjusting the final suspension to OD600 0.5. Then, 200 μL of the suspension was transferred to a 96‐well microplate, and 20 μL of ONPG (9 mg/mL) was added per well. O‐nitrophenol production was monitored by measuring absorbance at 420 nm using a spectrophotometer.

### 2.7 Superoxide Anions Generation by Bacterial Cells Under AgNPs Stress

Superoxide anion (O_2_˙^−^) generation by bacterial cells was measured using a modified nitroblue tetrazolium (NBT) reduction assay (Becerra and Albesa 2002). An overnight *S. aureus* culture was transferred to fresh TSB and incubated at 37°C for 6 h. After centrifugation and washing with sterile PBS, bacterial suspensions were resuspended in PBS and mixed with AgNPs at concentrations of 100, 75, 50, and 25 μg/mL in a 96‐well microplate. Controls included wells without nanoparticles and those containing only nanoparticles. After 12 h of incubation, 40 μL of 1 mM NBT was added to each well, and the plate was incubated in the dark for 30 min. The intensity of the blue color was measured at 560 nm, indicating NBT reduction to soluble formazan blue.

### 2.8 Morphology of *S. aureus* Cells Affected by AgNPs

The morphological changes in *S. aureus* exposed to AgNPs were examined using Field emission scanning electron microscopy (FE‐SEM) (Golding et al. 2016; Hisada et al. 2023). *S. aureus* was cultured in TSB, and the overnight culture was adjusted to 1 × 10^8^ CFU/mL. AgNPs were added at 75 μg/mL, and the culture was incubated at 37°C for 12 h. After centrifugation, the pellet was washed with sterile PBS, and bacterial cells were fixed with 2.5% glutaraldehyde for 3 h at 4°C. Fixed cells were dehydrated by incubating in increasing concentrations of ethanol (30%, 50%, 70%, 90%, 100%) for 10 min each. The samples were air‐dried, affixed to adhesive tape, and examined by FE‐SEM (GeminiSEM 500, ZEISS, Oberkochen, Germany).

### 2.9 Statistical Analysis

All experiments were performed in triplicate. Data was analyzed by one‐way ANOVA using SPSS (Chicago, IL, USA). Mean values were compared with Duncan's test at *p* < 0.05. A Student's *t*‐test was used to compare membrane permeability between nanoparticle‐treated and control groups.

## 3 Results

### 3.1 Characterization of AgNPs

**FIGURE 1:**
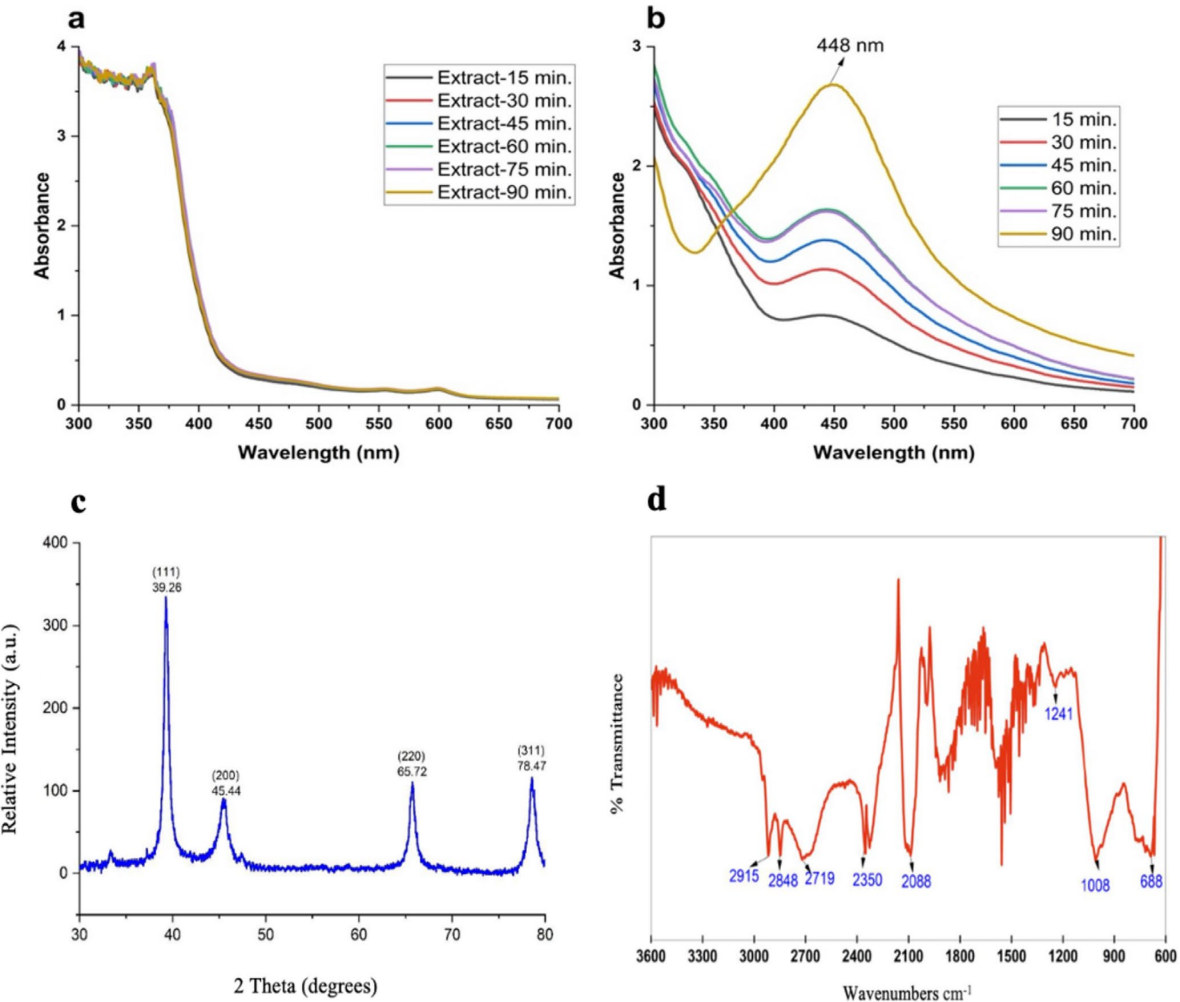
Structural characterization of biosynthesized AgNPs. (a) UV–Vis spectrum of the plant extract, (b) UV–Vis spectrum of AgNPs, (c) XRD pattern of AgNPs, (d) FTIR spectrum of AgNPs.

During AgNP synthesis, the reaction mixture changed to a dark brown‐black color, indicating AgNP formation. Nanoparticles were characterized at 15, 30, 45, 60, 75, and 90 min using UV‐Vis spectroscopy. The strongest peak appeared at 448 nm after 90 min, with nanoparticle formation beginning within 15 min. The surface plasmon resonance (SPR) bands were consistent with previous studies on AgNPs (Zaheer 2012). No visible change occurred in the plant extract, unlike in the nanoparticle samples (Figure 1a,b).

XRD analysis of AgNPs showed four distinct peaks at 2θ values of 39.26°, 45.44°, 65.72°, and 78.47° (Figure 1c), corresponding to the Bragg diffraction planes (111), (200), (220), and (310) of the face‐centered cubic (FCC) crystal structure of silver.

FTIR analysis was performed to identify functional groups involved in the reduction of silver nanoparticles. Absorption peaks at 2915, 2848, 2719, 2350, 2088, 1241, 1008, and 688 cm^−1^ were observed in AgNPs (Figure 1d). The peak at 688 cm^−1^ corresponds to out‐of‐plane C—H bending, and the weak band at 1241 cm^−1^ indicates C—O—C stretching in aromatic rings. Peaks at 1385 and 1445 cm^−1^ are attributed to C—H stretching. Bands between 1923 and 2000 cm^−1^ confirm the presence of an aromatic ring, while those at 2900–2990 cm^−1^ are linked to C—H stretching (Alahmad et al. 2022).

**FIGURE 2:**
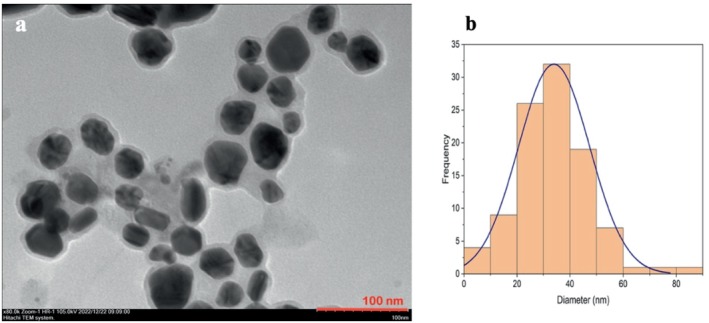
Morphological characterization of biosynthesized AgNPs. (a) Transmission electron micrograph of AgNPs, (b) particle size distribution of AgNPs.

TEM analysis of the synthesized nanoparticles, along with their size distributions, is shown in Figure 2. The TEM images were analyzed using ImageJ software, allowing for the measurement of the average diameters of 100 nanoparticles. The analysis revealed that the average nanoparticle diameter was approximately 35 ± 2.7 nm.

### 3.2 Antibacterial Activity of AgNPs

**FIGURE 3:**
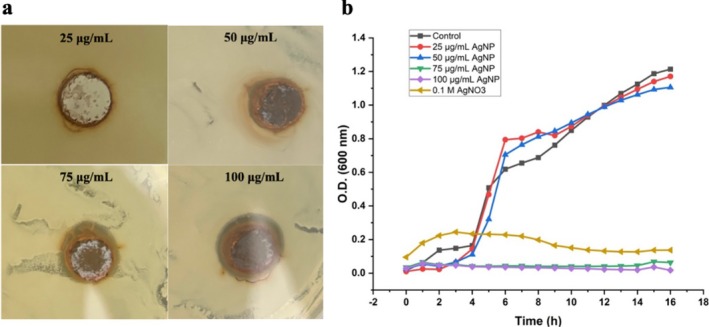
Antibacterial activity assays of biosynthesized AgNPs. (a) Inhibition zones against *S. aureus* , (b) growth curves of *S. aureus* treated with varying concentrations of AgNPs.

The MIC and MBC of silver nanoparticles (AgNPs) against *S. aureus* were 75 and 100 μg/mL, respectively. The well diffusion assay results in Figure 3a and Table 1 show that at the MIC of 75 μg/mL, the inhibition zone was 13 ± 0.13 mm. No inhibition zone was observed at 25 μg/mL, while higher concentrations of nanoparticles resulted in larger inhibition zones.

**TABLE 1:** Diameter of inhibition zones of nanoparticles against *S. aureus* . The results are provided as mean ± standard deviation (*n* = 3).

Nanoparticle concentrations	Inhibition zones (mm)
25 μg/mL	—
50 μg/mL	11 ± 0.2
75 μg/mL	13 ± 0.13
100 μg/mL	14 ± 0.12

The effects of AgNPs on *S. aureus* were assessed in a time‐ and concentration‐dependent manner using 96‐well microplates. In the control group (no nanoparticles), *S. aureus* entered the exponential growth phase after about 4 h, reaching an OD600 value of 1.2 after 16 h. In contrast, samples treated with AgNO_3_ at the concentration used for AgNP synthesis showed slight growth initially but stagnated after 6 h, with an OD600 stabilizing at approximately 0.4, suggesting that AgNO_3_ inhibits growth through a different mechanism, but less effectively. The study showed that AgNPs at a concentration of 100 μg/mL completely inhibited bacterial growth, while 75 μg/mL was the threshold concentration for inhibition (Figure 3b).

### 3.3 Antibiofilm Activity of AgNPs

**FIGURE 4:**
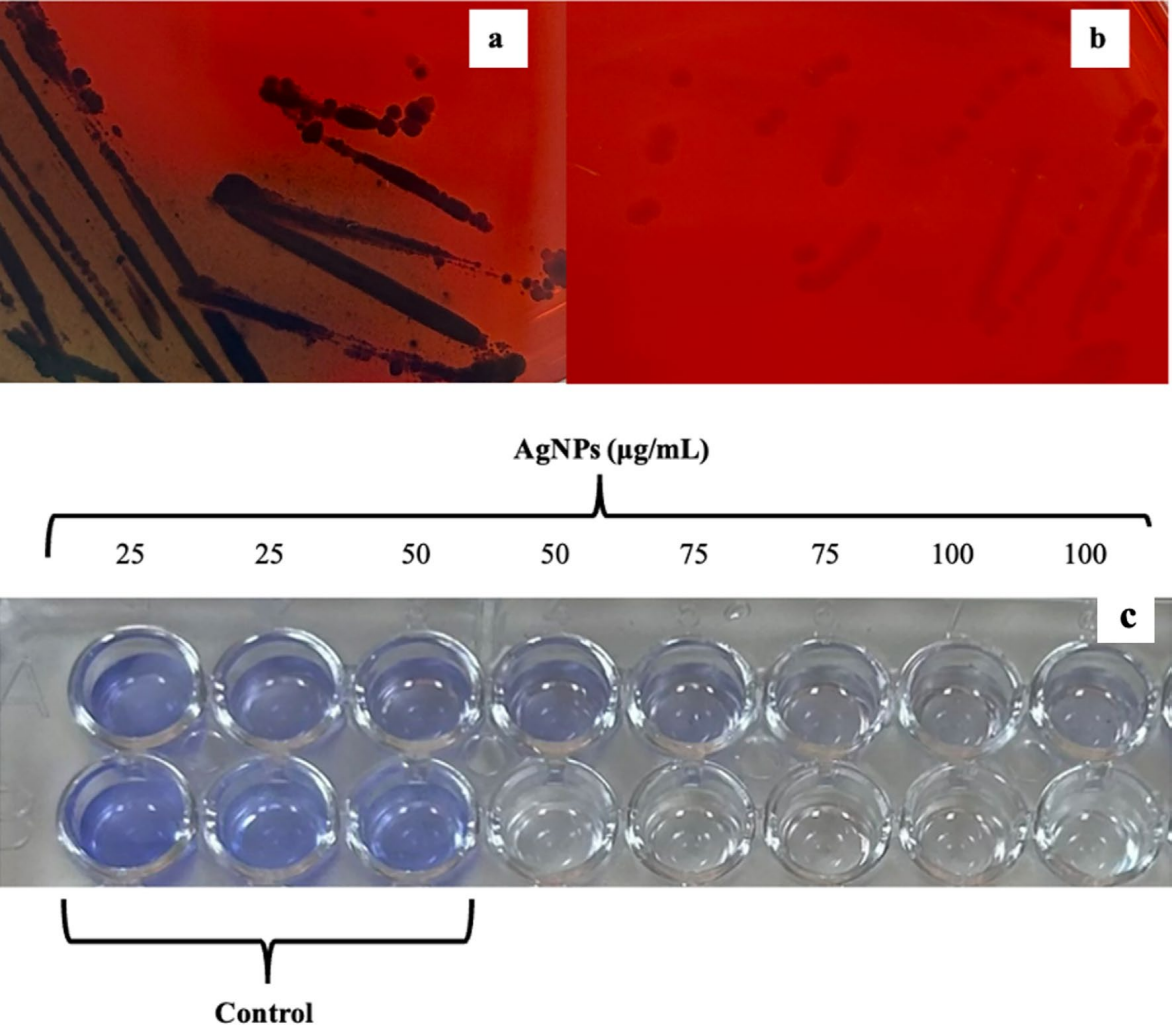
Biofilm formation and inhibition assays. (a, b) Detection of biofilm production with CRA for *S. aureus* (a) and *E. coli* (b), (c) quantification of biofilm inhibition activity of AgNPs on *S. aureus* in 96‐well microplate.

Biofilm production of *S. aureus* was confirmed using the CRA method (Figure 4a,b). Treatment with AgNPs at various concentrations significantly inhibited biofilm formation, with 47.25% ± 3.51% inhibition at 25 μg/mL. Table 2 shows biofilm inhibition percentages after 24 h exposure to AgNPs, ranging from 25 to 100 μg/mL. These results, depicted in Figure 4c, demonstrate that biofilm formation was inhibited in a concentration‐dependent manner by AgNPs.

**TABLE 2:** Inhibition effect of biosynthesized silver nanoparticles on biofilm formation of *S. aureus.* The results are provided as mean ± standard deviation (*n* = 3).

Nanoparticle concentrations	Biofilm inhibition (%)
25 μg/mL	47.25% ± 3.51%^c^
50 μg/mL	73.50% ± 3.00%^b^
75 μg/mL	83.20% ± 3.54%^a^
100 μg/mL	88.50% ± 3.84%^a^

*Note:* Means with the same letter are not significantly different (*p* > 0.05) different according to Duncan's test.

### 3.4 Effect of AgNPs on *S. aureus* Cellular Respiration

**FIGURE 5:**
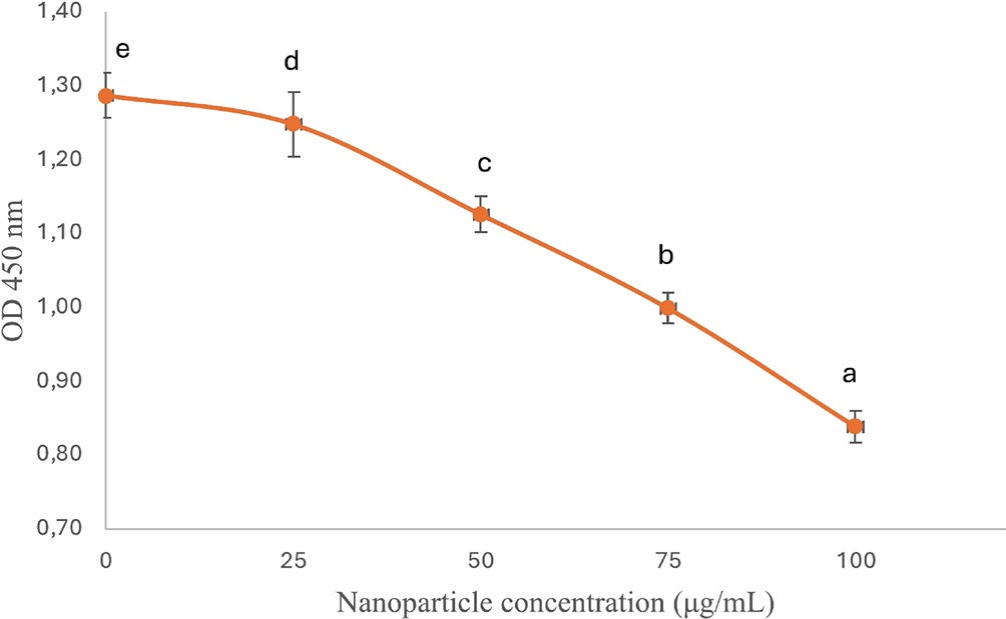
Inhibition of bacterial respiration dependent on AgNPs concentration. The letters indicate that each mean was different (*p* < 0.05) from any other according to Duncan's test.

The inhibition of *S. aureus* cellular respiration under nanoparticle stress was assessed using a TTC‐based spectroscopic method. As shown in Figure 5, increasing concentrations of silver nanoparticles led to a dose‐dependent decrease in OD values, reflecting the inhibition of cellular respiration. The control group (without nanoparticles) exhibited the highest OD of 1.288 ± 0.03, while at a 100 μg/mL nanoparticle concentration, the OD decreased below 0.9, demonstrating the significant impact of AgNPs on cellular activity.

### 3.5 Effect of AgNPs on *S. aureus* Cell Membrane Permeability

**FIGURE 6:**
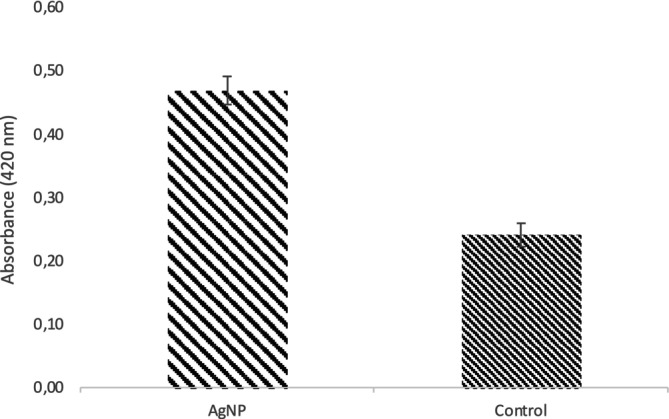
Effect of AgNPs on *S. aureus* cell membrane permeability. The difference between the two groups was statistically significant (*p* < 0.01).

Cell membranes are crucial for protecting the cell and regulating molecular transport (Watson 2015). To assess membrane permeability, β‐galactosidase activity was monitored by measuring the conversion of ONPG to O‐nitrophenol at 420 nm. The results showed that AgNPs significantly disrupted membrane integrity. In untreated *S. aureus* , absorbance was 0.24, while exposure to AgNPs increased it to 0.47 (Figure 6). This increase suggests that AgNPs enhance membrane permeability, allowing more β‐galactosidase to leak from the bacterial cells.

### 3.6 Superoxide Anions Generation by Bacterial Cells Under AgNPs Stress

**FIGURE 7:**
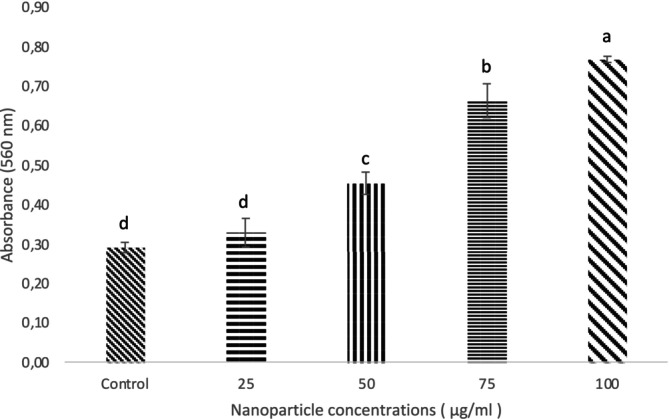
*S. aureus* superoxide anions generation under NP stress. Means with the same letter are not significantly (*p* > 0.05) different according to Duncan's test.

ROS, particularly superoxide anion (O_2_˙^−^), plays a major role in bacterial inhibition by damaging cellular components both inside and outside the cell. In this study, the generation of superoxide anions was assessed using the NBT assay. The results showed that AgNPs at a concentration of 100 μg/mL resulted in the highest absorbance, indicating that higher nanoparticle concentrations promote increased production of superoxide anions, thereby enhancing bacterial inactivation (Figure 7).

### 3.7 Morphology of *S. aureus* Cells Affected by AgNPs

**FIGURE 8:**
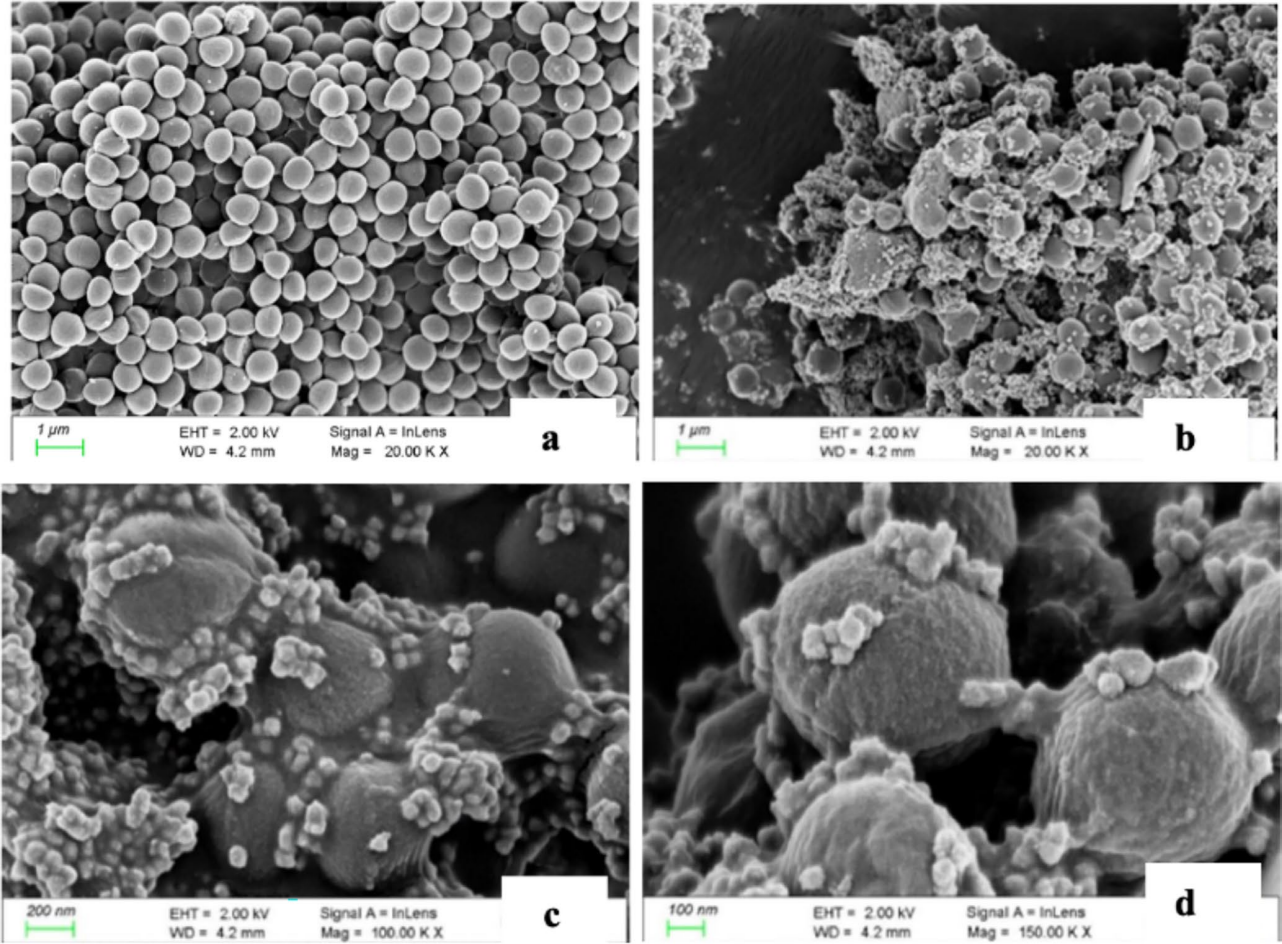
Scanning electron micrographs of *S. aureus* at different magnifications. (a) Grown without AgNPs; (b–d) grown with 75 μg/mL AgNPs.

FE‐SEM analysis of AgNP‐treated *S. aureus* cells (Figure 8) revealed distinct morphological changes compared to the control group. AgNP treatment (75 μg/mL) caused surface alterations, including voids, pits, and depressions, while untreated cells appeared smooth. The nanoparticles induced fragmentation and irregularities in the bacterial envelope. Additionally, AgNPs adsorbed onto the bacterial surface, forming aggregates of varying sizes.

## 4 Discussion

This study introduces an eco‐friendly approach for synthesizing silver nanoparticles (AgNPs) using an aqueous *H. perforatum* extract. The synthesized AgNPs displayed an absorption peak at 448 nm, aligning with previous studies (Rauf et al. 2025). XRD analysis confirmed their crystalline structure, with characteristic peaks at 2θ, in agreement with JCPDS data (Saravanakumar et al. 2015). FTIR spectra identified various functional groups, consistent with earlier findings (Alahmad et al. 2021, 2022). TEM images revealed that the AgNPs were consistent in size with those produced using other plant‐based synthesis methods (Gangal et al. 2025). The reduction and stabilization of the nanoparticles can be attributed to biomolecules like flavonoids, hypericins, and phenolic acids in the extract, which may enhance the AgNPs' antibacterial and antioxidant properties.

### 4.1 Antibacterial Activity of AgNPs

Numerous studies have demonstrated the successful synthesis of AgNPs from plant extracts and their antibacterial properties. Hosnedlova et al. (2022) synthesized silver nanoparticles (AgNPs) from *Lagerstroemia indica* , *Alstonia scholaris* , and *Aglaonema multifolium*, which exhibited antimicrobial activity against *S. aureus* with MIC values ranging from 15 to 20 μg/mL. In contrast, Ellis et al. (2018) observed that *Pseudomonas aeruginosa* developed resistance to AgNPs, whereas *S. aureus* remained susceptible. AgNPs synthesized from *Gardenia thailandica* leaf extract demonstrated antimicrobial activity against *S. aureus* with MIC values ranging from 4 to 64 μg/mL (Attallah et al. 2022). Similarly, AgNPs derived from grape seed extract resulted in an 85% reduction in *S. aureus* viability (Yaqubi et al. 2024). In this study, the biosynthesized AgNPs exhibited a MIC for *S. aureus* , along with a corresponding MBC, indicating strong antibacterial efficacy. These results align with findings from other plant‐derived AgNP studies, though some variation in effectiveness is noted. Such variability may arise from differences in synthesis methods, plant extract compositions, and nanoparticle properties, highlighting the complexity of AgNPs' antibacterial mechanisms.

The bacterial growth curve observed in this study demonstrated that increased concentrations of AgNPs significantly enhanced their time‐dependent bactericidal activity. This finding aligns with existing literature, which highlights the crucial role of AgNP concentration in determining their antimicrobial potency. Singh et al. (2021) reported that bacterial growth inhibition occurred within 4 h at AgNP concentrations of 100 μg/mL and 150 μg/mL, with notable cellular damage, including membrane fragmentation and morphological alterations. In a study by da Cunha et al. (2023), a 2 × MIC (15 μg/mL) concentration of Bio‐AgNPs inhibited *Staphylococcus epidermidis* within 6 h, while *S. aureus* strains were controlled after 24 h of treatment. At a 4 × MIC (30 μg/mL) concentration, bactericidal effects were observed across all strains within 6 h. Sun et al. (2016) demonstrated that both bare and quercetin‐conjugated AgNPs (30 μM) caused approximately a 60% reduction in cell viability for both *E. coli* and *S. aureus* .

### 4.2 Antibiofilm Activity of AgNPs

The study investigated the ability of *S. aureus* to develop biofilms after exposure to AgNPs, revealing a strong inhibitory effect of AgNPs on biofilm formation. This suggests the nanoparticles are highly effective even at moderate concentrations (Table 2). Similar findings were reported by Barabadi et al. (2021), where plant extract‐mediated AgNPs exhibited significant antibiofilm activity at concentrations ≥ 8 μg/mL. Subramaniyan et al. (2021) observed that *S. aureus* treated with 31.25 μM AgNPs showed weak adhesion and biofilm disintegration. Gupta et al. (2019) reported that AgNPs reduced biofilm formation in *S. aureus* and *P. aeruginosa* at concentrations of 125–250 μg/mL, with biofilm inhibition rates of 85% and 90%, respectively. Hussain et al. (2019) found that AgNPs reduced exopolysaccharide production by 61%–79% in both Gram‐positive and Gram‐negative bacteria, as well as decreased alginate production by 75.6% in *P. aeruginosa* . These findings align with our results, indicating that AgNPs are potent antibacterial agents with broad‐spectrum antibiofilm properties.

### 4.3 Impact of AgNPs on *S. aureus* Morphology and Cellular Functions

In this study, we examined the impact of AgNPs on cellular respiration, membrane permeability, cell morphology, and superoxide production in *S. aureus* to understand the underlying mechanisms driving their antimicrobial effects. While the precise mechanisms remain incompletely understood, AgNPs are thought to exert antimicrobial activity through three main pathways: (1) inducing cell lysis via interactions with the peptidoglycan cell wall and membrane, (2) disrupting protein synthesis by binding to bacterial proteins, and (3) inhibiting DNA replication through interactions with bacterial cytoplasmic DNA (Song and Ge 2019). Additional proposed mechanisms include metal ion toxicity resulting from the dissolution of metals from nanoparticle surfaces and oxidative stress generated by ROS on these surfaces (Besinis et al. 2014). It has been shown that the positive charge of Ag^+^ ions in silver nanoparticles enhances their antimicrobial activity by facilitating electrostatic interactions between the negatively charged bacterial membrane and the positively charged nanoparticles (Kim et al. 2007).

During bacterial respiration, cytochrome systems facilitate the reduction of TTC to triphenyl formazan (TPF), a red compound that serves as an indicator of cellular metabolic activity (Erdem et al. 2015). Our findings reveal a significant reduction in TPF formation as the concentration of AgNPs increases, suggesting that AgNPs disrupt bacterial respiration (Figure 5). This decline in TPF formation implies that AgNPs inhibit the bacterial cell membrane's ability to maintain a negative redox potential, thereby impeding TTC reduction. These results align with previous studies, including Qais and Ahmad (2018), who demonstrated that silver nanoparticles synthesized from *Withania somnifera* significantly reduced *S. aureus* respiration in a concentration‐dependent manner, with more than a threefold decrease in respiration observed in the presence of WS‐AgNPs. The antimicrobial action of AgNPs is thought to involve interactions with respiratory enzyme sulfhydryl or disulfide groups, leading to disruption of the respiratory chain and alterations in membrane permeability (Egger et al. 2009). Our study further corroborates this mechanism, highlighting the significant role of AgNPs in modifying bacterial membrane permeability as a key contributor to their antimicrobial effect (Figure 6).

β‐galactosidase activity, a widely accepted marker for membrane permeability, was assessed by monitoring the hydrolysis of ONPG to O‐nitrophenol, with absorbance measured at 420 nm (Han et al. 2013). The data revealed a marked increase in absorbance—approximately twofold—between the control and AgNP‐treated cells, indicating substantial membrane disruption induced by the nanoparticles. This effect may be attributed to the adsorption of AgNPs onto the bacterial cell membrane, with some nanoparticles penetrating the cytoplasm, subsequently interacting with intracellular structures and causing the leakage of cellular contents (Sun et al. 2016; Zhang et al. 2018). Such membrane disruption has been observed in *S. aureus* , where treatment with AgNPs resulted in the release of intracellular material, leading to collapsed, vacuolated cells. These findings are consistent with research suggesting that the binding of nanoparticles to membrane proteins can result in the inactivation of membrane‐associated enzymes, thereby increasing membrane permeability and facilitating the efflux of vital intracellular components (Diaz‐Visurraga et al. 2010).

AgNPs can cause significant disruptions to the bacterial membrane, leading to the formation of pits and voids, and ultimately resulting in cell lysis (Yun et al. 2013). In our study, FE‐SEM analysis (Figure 8) revealed an increase in surface roughness in AgNP‐treated *S. aureus* cells compared to control cells, suggesting membrane damage. The accumulation of AgNPs on the bacterial surface likely contributes to decreased viability, corroborating findings from Shaaban et al. (2023), who observed similar morphological changes and pore formation on *S. aureus* after AgNP treatment. Ahmed et al. (2020) also reported similar effects on bacterial cells treated with AgNPs and ZnO‐NPs, showing voids, pits, and fragmented cell walls. The disruption of membrane integrity appears to result from AgNPs binding to the cell membrane and penetrating the cytoplasm, causing leakage of cellular contents (Zhang et al. 2018). This disruption is thought to be due to the inactivation of membrane‐bound proteins, which increases permeability and facilitates the efflux of intracellular components, further compromising bacterial integrity (Diaz‐Visurraga et al. 2010).

Nanoparticles exhibit antimicrobial activity by generating ROS, such as superoxide anions (O_2_˙^−^), through disruption of cellular functions like the respiratory chain (Kashyap et al. 2024). In our study, NBT assay results showed that AgNPs at 100 μg/mL produced the highest absorbance, indicating increased superoxide anion generation and enhanced bacterial inactivation (Figure 7). This finding aligns with Yuan et al. (2017), who reported a twofold increase in ROS production in *P. aeruginosa* and *S. aureus* following AgNP treatment. Similarly, Quinteros et al. (2016) observed ROS induction in *E. coli* , *S. aureus* , and *P. aeruginosa* , triggering oxidative stress. Ahmed et al. (2020) confirmed that higher concentrations of AgNPs and ZnO‐NPs correlate with increased ROS production.

## 5 Conclusions

The present study concluded by successfully demonstrating the environmentally friendly synthesis of silver nanoparticles (AgNPs) using extract from *H. perforatum* , followed by thorough characterization using a variety of analytical methods. The antibacterial activities of the synthesized AgNPs were substantiated through FE‐SEM analysis, which highlighted their efficacy in disrupting cellular integrity. Furthermore, the investigation into the effects of AgNPs on cellular respiration, membrane permeability, cell morphology, and superoxide production in *S. aureus* illustrated their multifaceted mechanism of action. The findings reveal that AgNPs possess significant antimicrobial properties, including a remarkable ability to inhibit bacterial biofilm formation and disrupt essential cellular functions. The bactericidal effect of AgNPs was notably concentration‐dependent, with higher concentrations gradually corresponding to improved antibacterial activity. These findings confirm the effectiveness of bioengineered metallic nanoparticles, particularly AgNPs, in targeting drug‐resistant bacteria. However, challenges remain regarding biocompatibility and toxicity at higher concentrations, as well as the scalability and reproducibility of synthesis methods (Nayak et al. 2023). Despite these challenges, AgNPs hold great potential as therapeutic agents, with ongoing research into their molecular mechanisms crucial for optimizing their future use in nanomedicine.

## Author Contributions

**Canan Sevinc‐Sasmaz:** methodology, investigation, visualization, writing – review and editing, data curation, writing – original draft. **Fatih Erci:** writing – review and editing, project administration, resources, conceptualization. **Emrah Torlak:** supervision, writing – review and editing, formal analysis. **Mustafa Yöntem:** methodology.

## Conflicts of Interest

The authors declare no conflicts of interest.

## Data Availability

All data generated or analyzed during this study are included in the figures and tables of this published article.
